# Proprotein convertase subtilisin/kexin type 9: a promising marker of cardiovascular risk in post-menopausal diabetic women in primary prevention

**DOI:** 10.3389/fmed.2025.1521344

**Published:** 2025-03-12

**Authors:** Michelangelo Rottura, Maria Antonietta Barbieri, Carmine Siniscalchi, Pierpaolo Di Micco, Selene Francesca Anna Drago, Marianna Gigliotti De Fazio, Arrigo Francesco Giuseppe Cicero, Federica Fogacci, Giuseppe Armentaro, Angela Sciacqua, Vincenzo Arcoraci, Natasha Irrera, Egidio Imbalzano

**Affiliations:** ^1^Department of Clinical and Experimental Medicine, University of Messina, Messina, Italy; ^2^Department of Medicine and Surgery, University of Parma, Parma, Italy; ^3^AFO Medicina PO Santa Maria delle Grazie, Pozzuoli Naples Hospital, Naples, Italy; ^4^IRCSS Policlinico S. Orsola-Malpighi, Hypertension and Cardiovascular Risk Research Center, DIMEC, University of Bologna, Bologna, Italy; ^5^Department of Medical and Surgical Sciences, University Magna Græcia of Catanzaro, Catanzaro, Italy

**Keywords:** Proprotein convertase subtilisin/kexin type 9 (PCSK9), cardiovascular risk, post-menopausal women, diabetes, pulse wave velocity

## Abstract

**Background and aims:**

Proprotein convertase subtilisin/kexin type 9 (PCSK9) increases circulating LDL levels and cardiovascular disease (CVD) risk; its levels may be related to the dysregulation of glycemic control and may be affected by estrogens. The aim of this study was to assess factors related to PCSK9 levels, and to evaluate the correlation between PCSK9 levels and CV parameters in post-menopausal diabetic women in primary prevention.

**Methods:**

Generalized linear models (GLM) were adopted to evaluate predictors of PCSK9 levels as well as factors related to CV outcomes, such as pulse wave velocity (PWV), pulse pressure (PP), and augmentation index (AI).

**Results:**

A total of 135 post-menopausal diabetic women, with a median (Q1-Q3) serum PCSK9 levels of 370.3 (344.0–409.4) ng/ml were enrolled. Apolipoprotein B values resulted an independent predictor of PCSK9 levels (*B* = 1.023; *p <* 0.001). However, LDL values were inversely related to PCSK9 levels (*B* = −0.578; *p <* 0.001). PCSK9 levels influenced PWV (*B* = 0.010; *p =* 0.010), but did not influence other CV outcomes.

**Conclusion:**

ApoB and LDL may influence PCSK9 levels and PCSK9 directly influence PWV in post-menopausal diabetic women in primary prevention. Therefore, the relationship between PCSK9 and primary prevention cannot be excluded, thus highlighting its role as biomarker of CV risk.

## 1 Introduction

Proprotein convertase subtilisin/kexin type 9 (PCSK9) is a circulating serine protease widely expressed in the liver that is involved in the regulation of blood cholesterol hemostasis and low-density lipoprotein (LDL) receptor (LDLR) degradation on hepatocytes, thus consequently inducing increased circulating LDL levels (1). This increase may represent an important risk factor for hypercholesterolemia, cardiovascular diseases (CVD), atherosclerosis, coronary artery disease (CAD) and stroke (2).

Interest in the role of PCSK9 in the regulation of LDL metabolism and in the pathogenesis of related diseases is increasing over the years, and previous studies have already demonstrated that PCSK9 gain-of-function mutations may be associated with autosomal dominant hypercholesterolemia and premature atherosclerosis (3), whereas loss-of-function mutations may lead to LDL level reduction and therefore may be protective against CV events (4). Although the role of PCSK9 in the liver is well defined, extrahepatic action was also observed; in fact, high levels of PCSK9 were detected in the gastrointestinal tract and in the kidney, macrophages, endothelial cells (ECs) and vascular smooth muscle cells (VSMCs). Preclinical studies have shown that increased PCSK9 levels may induce proinflammatory gene expression and apoptosis, thus promoting endothelial dysfunction far beyond LDL metabolism regulation. As a result, an increase in PCSK9 may play a direct role in the progression of atherosclerotic lesions, whereas its inhibition may be protective, with additional pleiotropic effects (5, 6). In patients with ACS and CAD, elevated plasma PCSK9 levels were found to be independently linked to inflammatory indicators such fibrinogen levels, high sensitivity C-reactive protein (hs-CRP) levels, and white blood cell count (WBCC) (**7**). Furthermore, it has been discovered that PCSK9 increases the synthesis of proinflammatory cytokines; for example, the PCSK9-induced enhancement of the inflammatory response may be mediated via the stimulation of the TLR4/NF-κB signaling pathway (8). Moreover, PCSK9 might seem to have an antithrombotic effect through platelet function and blood coagulation modulation (9). Data on the physiological role of PCSK9 in glucose metabolism and renal function are controversial, but a significant variable association between plasma levels of PCSK9 and dysregulation of glycemic control or worsening of kidney impairment cannot be excluded (5). Furthermore, PCSK9 concentration may be influenced by estrogens: high levels may decrease PCSK9 concentration with the consequent increase of LDLRs expression in liver (10). In fact, a significant increase in PCSK9 was observed in post-menopausal women as a consequence of the decrease in estrogen levels (11).

Circulating PCSK9 appears to be produced mainly by the liver, and its expression is regulated by numerous factors, such as thyroid hormone and thyroid replace therapy (12), diet (13), endogenous insulin and therapeutic exogenous insulin (14), resistin (15), the diurnal rhythm (16), various cholesterol-lowering drugs (17) and exercise (18). It seems that sex could modify the effects of extrinsic and intrinsic factors on the PCSK9 concentration.

The relationship between traditional CV risk factors and CV biomarkers has been well studied, but the impact of PCSK9 levels on CV risk has yet to be implemented, especially in the context of primary prevention. In particular, the evaluation of surrogate and prognostic markers of CV risk pointed out a possible correlation between arterial stiffness measured by pulse wave velocity (PWV) and atherosclerosis (19) considering the analogous underlying mechanisms in plaque formation and arterial stiffening, PCSK9 accumulation in atherosclerotic plaques could also be associated with arterial wall remodeling (20). In this context, pulse pressure (PP) may also be considered a risk factor for arterial stiffness and assumes a predictive role in CVD mortality (21, 22). A negative association between PCSK9 and PP was described in normotensive female patients (23), but recently, a correlation with high levels of PCSK9 values and PP was detected in diabetic patients (24). Additionally, the augmentation index (AI), which is used as a measure of wave reflection and arterial stiffness, showed a significant linear positive correlation with PCSK9 levels both in obese patients and in patients with familial dyslipidemias (25, 26).

Different studies have already evaluated the role of PCSK9 in CV endpoints only according to sex or a specific disease in secondary prevention (24, 27). However, primary prevention in the context of CVD is an important tool for healthcare to modify potential risk factors as soon as possible and reduce the risk of CV events, especially in post-menopausal women. Therefore, the aim of the present study was to observe the correlation between PCSK9 levels and CV parameters in post-menopausal diabetic women in primary prevention. Factors associated with PCSK9 levels in the same cohort of patients were also investigated as secondary objective.

## 2 Materials and methods

### 2.1 Study design and data collection

An observational study was conducted on post-menopausal diabetic women in primary prevention, monitored by the Internal Medicine Unit of the University Hospital of Messina, to evaluate PCSK9 levels and the possible correlation with CV parameters as well as factors associated with PCSK9 concentration. All computerized medical records were analyzed from May 2021 to October 2022 for each patient and collected in a dedicated database that includes information on sociodemographic characteristics, clinical and laboratory parameters, comorbidities, and drug therapies. Comorbidities were codified according to the International Classification of Diseases 9th Revision (ICD-9-CM), while drugs were classified according to the ATC classification. An encrypted code was used for each patient in accordance with the law on privacy. The study was conducted in compliance with the guidelines of the Declaration of Helsinki and approved by the Local Ethics Committee of Messina (protocol number #5020).

In detail, post-menopausal women with at least one registered PCSK9 value were identified from the dataset. The serum concentrations of PCSK9 were measured by using commercially available enzyme-linked immunosorbent assay (ELISA) kits according to the instructions reported by the manufacturer. All the samples were evaluated in duplicate, and the obtained results were interpolated with the respective standard curves.

Women were classified by menopausal age in the following menopause stage: early-onset menopause (age < 45 years), normal-onset menopause (45–55 years), and late-onset menopause (>55 years). The following characteristics and comorbidities were evaluated: age, BMI and smoking habits. All diagnostic instrumental and laboratory tests, such as DBP, SBP, total cholesterol, LDL, HDL, triglycerides, ApoB, and FPG values, were also collected. Moreover, the estimated glomerular filtration rate (eGFR) was calculated using the CKD-EPI formula.

All CV function measurements were calculated: PP, defined as the difference between SBP and DBP (28); PWV, defined as the distance covered by the pulse wave divided by the time that the pulse wave needs to cover that distance (m/s) (29); AI, defined as the ratio between the second (P2) and first (P1) systolic peak pressure caused by the reflected wave to PP (30).

### 2.2 Data analysis

Descriptive analyses were performed to evaluate clinical and demographics characteristics of patients stratified according to PCSK9 concentration quartile ranges and expressed as medians (first and third quartile, Q1-Q3) for continuous variables and absolute values (percentages) for categorical variables. Moreover, PCSK9 level distributions were evaluated by menopause duration and menopause age, and stratified by menopausal stage. The Pearson chi-square test and Kruskal–Wallis H test were carried out to compare categorical variables and continuous variables, respectively. Univariate correlations were analyzed with Spearman’s rank correlation coefficient.

The Kolmogorov–Smirnov standardized test confirmed that PCSK9 levels as well as log transformation of PCSK9 (logPCSK9) were not normally distributed. Therefore, generalized linear models (GLM) were adopted to evaluate factors associated with PCSK9 concentration as well as to identify factors correlated with CV outcomes (PWV, PP, and AI), including PCSK9 levels as covariate.

A *p* value <0.05 was considered statistically significant for all analyses performed with SPSS version 29.0 (IBM Corp., SPSS Statistics).

## 3 Results

### 3.1 Baseline characteristics and cardiovascular measures

**Figure 1:**
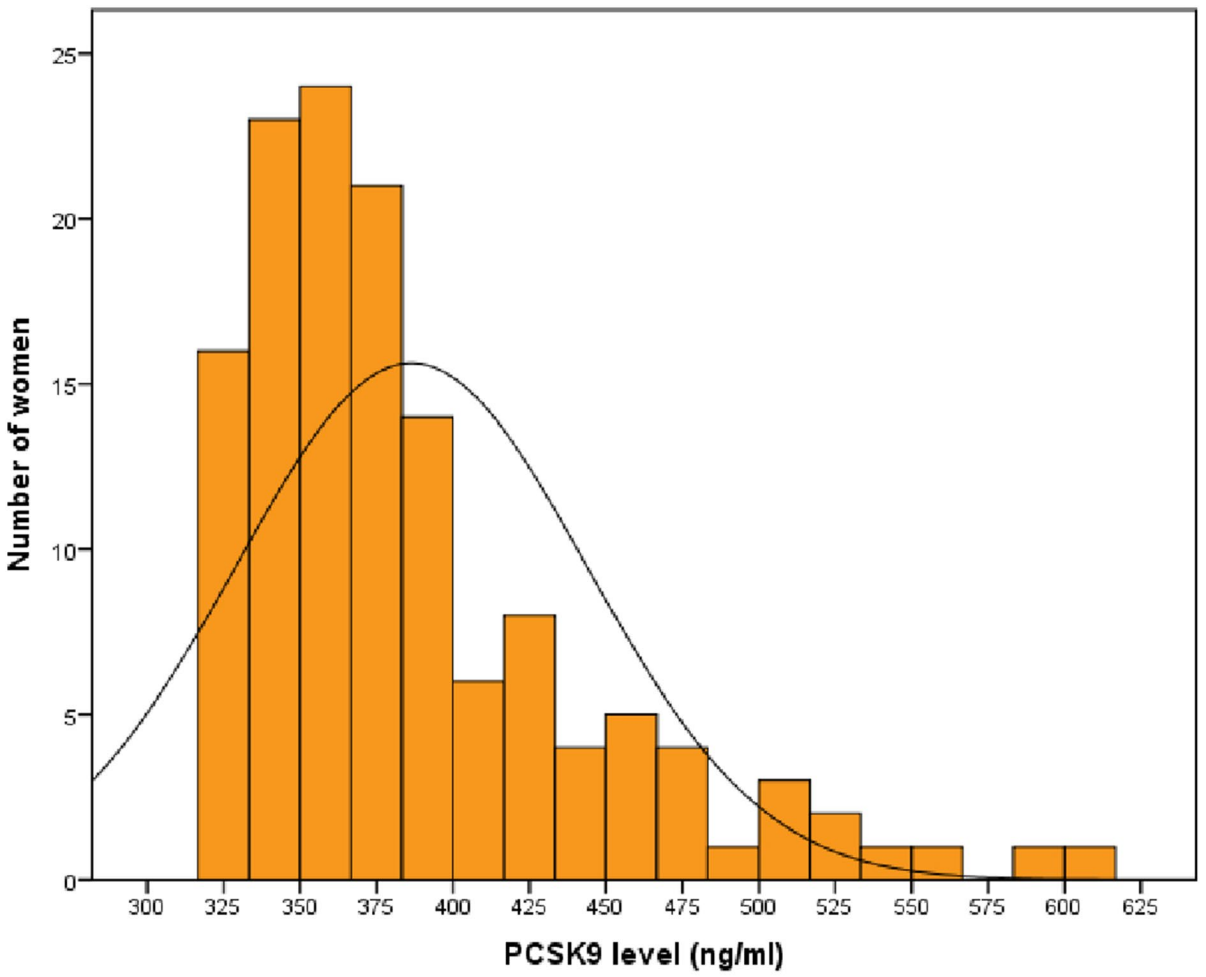
Distribution of PCSK9 values in post-menopausal women with diabetes. PCSK9: proprotein convertase subtilisin/kexin type 9.

A total of 135 post-menopausal diabetic women in primary prevention were enrolled in this study. Women had a median (Q1-Q3) age of 65 (60–75) years, and 23 (17.0%) were habitual smokers. Median (Q1-Q3) serum PCSK9 levels were 370.3 (344.0–409.4) ng/ml (Figure 1).

The median (Q1-Q3) clinical and laboratory parameters were as follows: LDL, 151.6 (123.2–181.4) mg/dl; high-density lipoprotein (HDL), 53.0 (44.0–61.0) mg/dl; triglycerides, 113.0 (87.0–160.0) mg/dl; total cholesterol, 229.0 (205.0–258.0) mg/dl; fasting plasma glucose (FPG), 91.0 (84.0–100.0) mg/dl; and apolipoprotein B (ApoB), 98.0 (85.0–120.0) mg/dl. Moreover, the systolic blood pressure (SBP)/ diastolic blood pressure (DBP) [median (Q1-Q3)] was 148/72 (133/66–161/78) mmHg, body mass index (BMI) 26.8 (23.6–30.0) Kg/m^2^, and Chronic Kidney Disease Epidemiology Collaboration (CKD-EPI) 63.8 (55.8–71.8) ml/min. Median (Q1-Q3) CV outcomes were: PWV 9.8 (8.3–11.3) m/s, PP 76 (64–87) mmHg, AI 29 (24–34) %. Significant differences were observed for LDL, ApoB and cholesterol levels among PCSK9 quartile ranges (in order: *p* = 0.012, *p* = 0.009, and *p* = 0.013). The analysis of CV outcomes showed no significant differences among PCSK9 quartiles (Table 1).

**Table 1:** Baseline characteristics of post-menopausal women stratified by PCSK9 levels.

Variables	Q1 *N* = 33	Q2 *N* = 34	Q3 *N* = 33	Q4 *N* = 35	*P* value
Age	64 (58–71)	71 (58–78)	65 (60–73)	65 (61–76)	0.378
Smoking	6 (18.2)	5 (14.7)	8 (24.2)	4 (11.4)	0.541
BMI	27.0 (23.5–29.1)	25.9 (23.3–29.8)	26.0 (22.9–31.0)	27.1 (24.2–29.7)	0.920
PWV	10.1 (8.3–11.5)	9.3 (8.7–10.6)	9.0 (7.8–10.7)	10.5 (8.9–13.5)	0.079
PP	76 (63–93)	74 (62–86)	77 (63–86)	75 (66–85)	0.974
AI	29.0 (23.5–35.0)	26.0 (23.0–33.0)	32.0 (25.5–36.0)	29.0 (26.0–32.0)	0.113
Systolic pressure	151 (133–165)	145 (131–168)	146 (132–157)	149 (139–157)	0.910
Diastolic pressure	73 (65–80)	75 (67–80)	69 (65–76)	72 (70–77)	0.369
Cholesterol	229.0 (207.5–255.0)	254.0 (218.8–278.0)	212.0 (174.0–249.5)	221.0 (196.0–248.0)	0.013
HDL	48.0 (40.0–64.5)	55.0 (44.3–63.5)	54.0 (45.0–61.0)	52.0 (43.0–59.0)	0.828
Triglycerides	117.0 (8.0–159.5)	136.0 (90.8–200.0)	106.0 (67.0–135.0)	119.0 (88.0–160.0)	0.160
LDL	155.8 (128.9–180.4)	165.0 (147.9–191.3)	135.6 (111.4–167.5)	143.2 (117.6–173.0)	0.012
FPG	90.0 (85.5–100.5)	91.0 (82.0–97.5)	94.0 (82.0–103.5)	92.0 (86.0–101.0)	0.931
ApoB	93.0 (85.0–105.0)	101.5 (84.5–120.3)	90.0 (78.5–106.5)	112.0 (96.0–129.0)	0.009
CKD EPI	64.1 (56.9–73.8)	64.1 (55.4–72.3)	65.4 (52.9–69.8)	61.4 (54.1–72.2)	0.867

^*^Pearson Chi-Square test for categorical variables and Kruskal-Wallis H Test for continuous variables. AI, augmentation index; ApoB, apolipoprotein B; BMI, body mass index; CKD EPI, chronic kidney disease Epidemiology Collaboration; FPG, fasting plasma glucose; HDL, high density lipoprotein; LDL, low density lipoprotein; PCSK9, proprotein convertase subtilisin/Kexin-type 9; PP, pulse pressure; PWV, pulse wave velocity.

**Figure 2:**
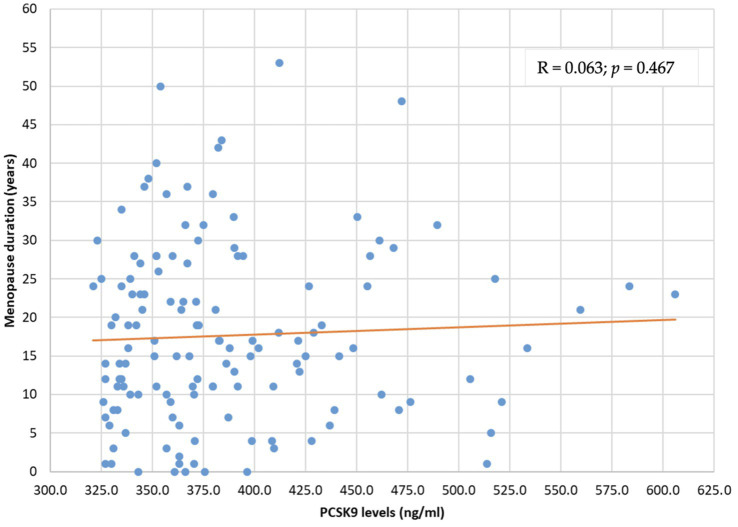
PCSK9 level distribution by menopause duration (years). PCSK9: proprotein convertase subtilisin/kexin type 9.

**Figure 3:**
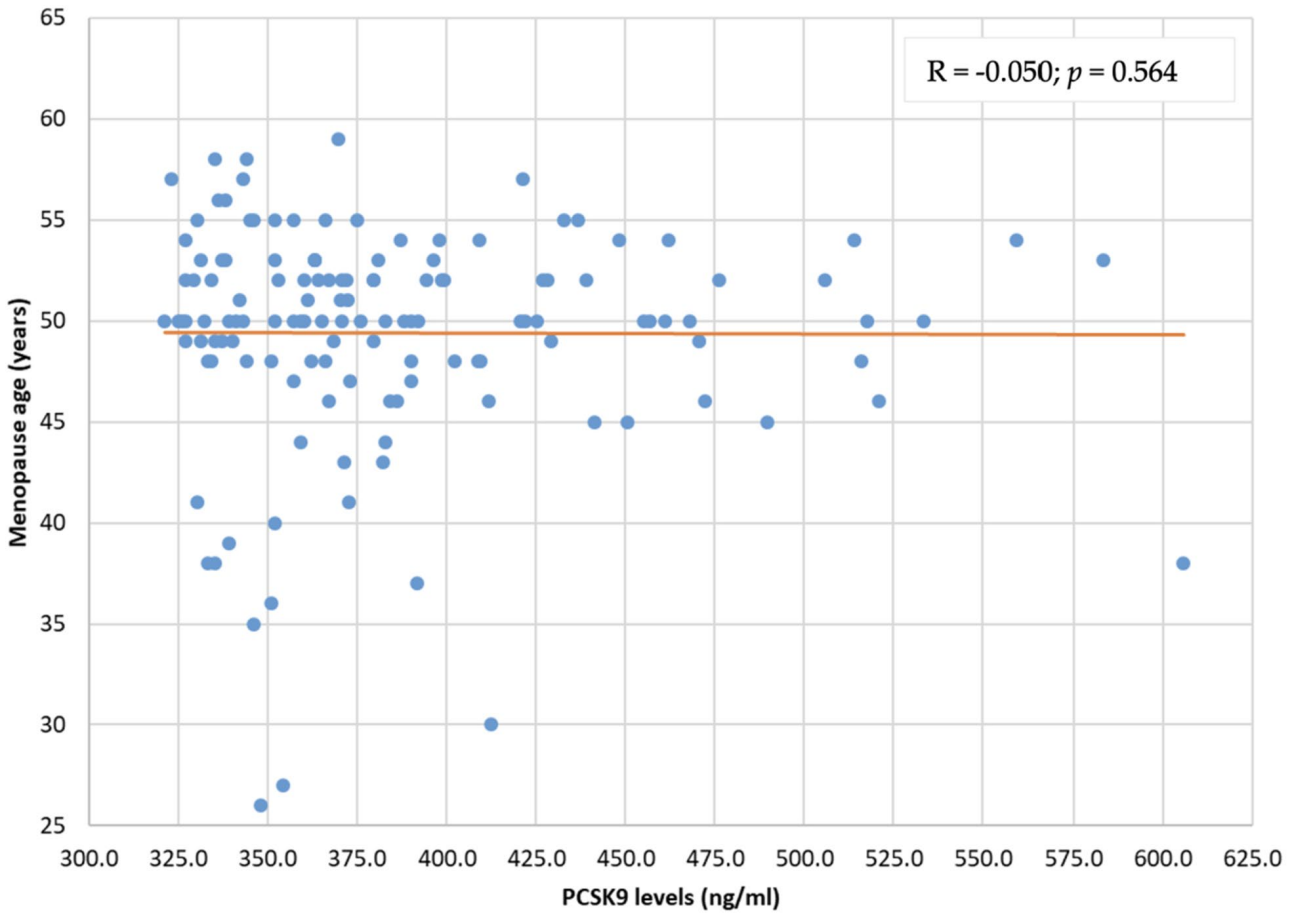
PCSK9 level distribution by menopause age (years). PCSK9: proprotein convertase subtilisin/kexin type 9.

The distribution of PCSK9 levels was not related to menopause duration (R = 0.063; *p* = 0.467) and menopause age (*R* = −0.050; *p* = 0.564) (Figures 2, 3).

**Figure 4:**
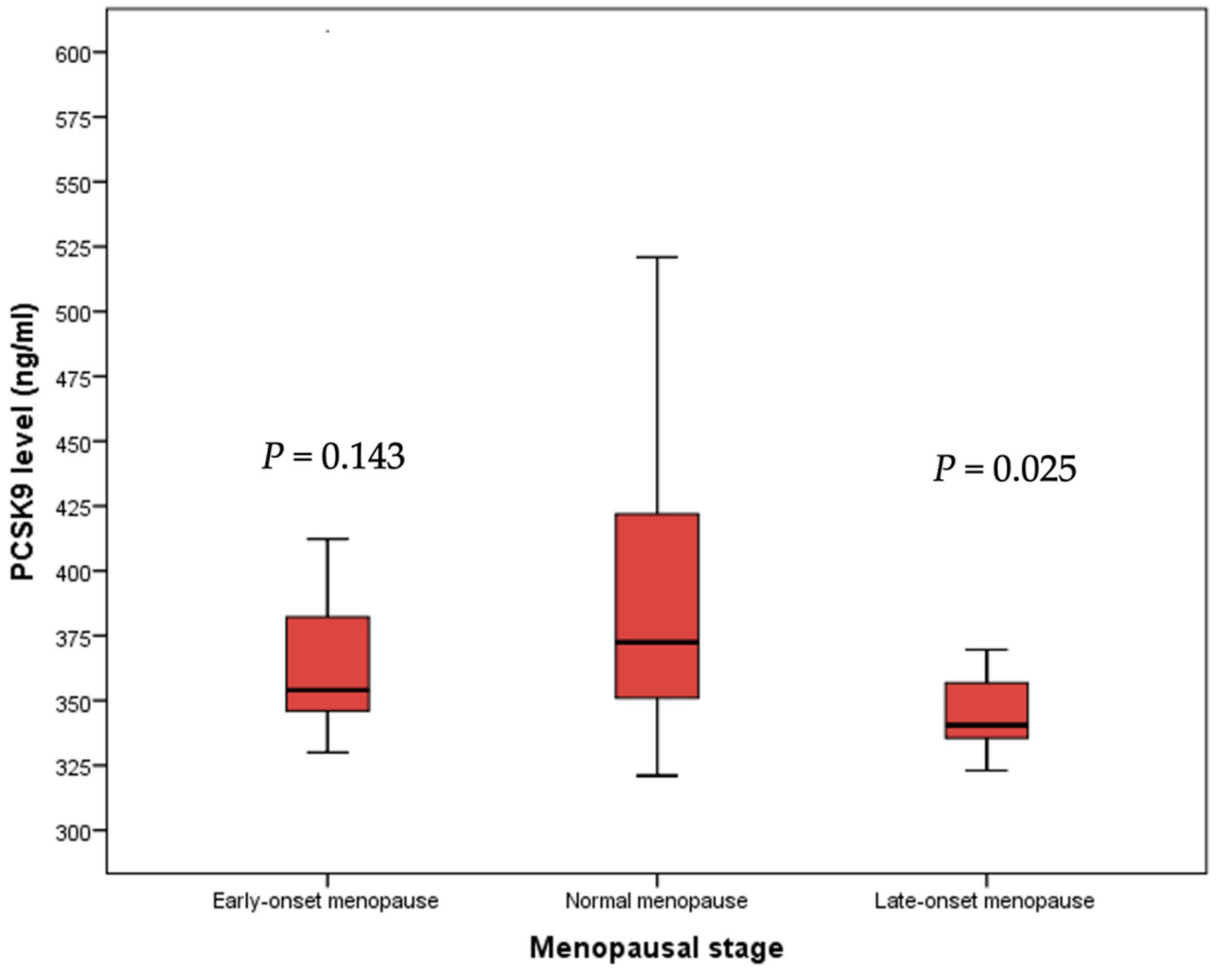
Median PCSK9 levels stratified by menopausal stage. PCSK9: proprotein convertase subtilisin/kexin type 9.

However, a significant reduction of PCSK9 levels was detected in late-onset menopause women when compared to early- and normal- onset menopause women (*p* = 0.025) (Figure 4).

### 3.2 Relationship between risk factors and PCSK9 levels

GLM model indicated that ApoB values were an independent predictor of PCSK9 concentration (*B* = 1.023; *p* < 0.001). However, LDL values were inversely related to PCSK9 concentration (*B* = −0.578; *p* < 0.001), as well as late-onset menopause stage (*B* = −44.798; *p* = 0.022) (Table 2).

**Table 2:** Predictive factors of PCSK9.

Variables	PCSK9	
	B	*P* value
Age	0.380	0.429
Smoking	−7.267	0.576
BMI	1.291	0.266
Systolic pressure	0.040	0.891
Diastolic pressure	0.073	0.899
HDL	−0.363	0.268
Triglycerides	−0.095	0.272
LDL	−0.578	<0.001
FPG	−0.093	0.814
ApoB	1.023	<0.001
Normal menopause	Ref	
Early-onset menopause	−10.312	0.459
Late-onset menopause	−44.798	0.022

ApoB, apolipoprotein B; BMI, body mass index; CKD EPI, chronic kidney disease Epidemiology Collaboration; FPG, fasting plasma glucose; HDL, high density lipoprotein; LDL, low density lipoprotein; PCSK9, proprotein convertase subtilisin/Kexin-type 9.

### 3.3 Formatting of mathematical components

PCSK9 levels, FPG, and SBP influenced PWV (in order *B* = 0.010; *p* = 0.010, *B* = 0.037; *p* = 0,037, and *B* = 0.051; *p* < 0.001) (Table 3). Conversely, BMI, HDL, and DBP were inversely associated with PWV (in order *B* = −0.154; *p* = 0.003, *B* = −0.039; *p* = 0.009, and *B* = −0.060; *p* = 0.022). Moreover, SBP positively influenced PP (*B* = 0.014; *p* < 0.001), while DBP inversely influenced PP (*B* = −0.014; *p* < 0.001). The only predictive factor that inversely influenced AI was DBP (*B* = −0.256; *p* = 0.004). Moreover, PCSK9 levels not influenced PP and AI (*B* = −0.007; *p* = 0.580 and *B* = 0.000; *p* = 0.885, respectively) (Table 3).

**Table 3:** Correlations between risk factors and cardiovascular endpoints.

Variables	PWV		AI		PP	
	B	*P* value	B	*P* value	B	*P* value
Age	0.034	0.122	−0.078	0.291	0.000	0.884
Smoking	−0.062	0.915	−0.810	0.684	0.012	0.280
BMI	−0.154	0.003	0.222	0.214	0.000	0.829
Systolic pressure	0.051	<0.001	0.094	0.036	0.014	<0.001
Diastolic pressure	−0.060	0.022	−0.256	0.004	−0.014	<0.001
HDL	−0.039	0.009	0.005	0.918	0.000	0.264
Triglycerides	0.001	0.841	−0.006	0.677	0.000	0.178
LDL	−0.010	0.159	−0.022	0.349	0.000	0.568
FPG	0.037	0.037	0.068	0.262	0.000	0.553
ApoB	0.010	0.404	0.046	0.270	0.000	0.463
PCSK9	0.010	0.010	−0.007	0.580	0.000	0.885
Normal menopause	Ref		Ref		Ref	
Early-onset menopause	−0.467	0.459	1.734	0.417	0.002	0.898
Late-onset menopause	−0.643	0.476	−2.070	0.499	0.004	0.817

AI, augmentation index; ApoB, apolipoprotein B; BMI, body mass index; CKD EPI, chronic kidney disease Epidemiology Collaboration; HDL, high density lipoprotein; LDL, low density lipoprotein; PCSK9, proprotein convertase subtilisin/Kexin-type 9; PP, pulse pressure; PWV, pulse wave velocity.

## 4 Discussion

Proprotein convertase subtilisin kexin type 9 (PCSK9) is a proprotein convertase that increases plasma low-density lipoprotein cholesterol (LDL-C) levels by triggering the degradation of LDL receptors (LDLRs). PCSK9 is linked to coronary plaque inflammation and has direct atherosclerotic effects on the vascular wall in addition to controlling the amount of LDL-C in the blood.

The results of our study show that diabetic women with late-onset menopause have a reduction of PCSK9 levels; furthermore, PCSK9 concentration was inversely correlated with aortic stiffness, indirectly measured with PWV, thus letting us to hypothesize that PCSK9 might represent a predictive marker of arterial stiffness and CV risk in this specific subset of patients.

Predictive factors of PCSK9 levels and PCSK9 influence on CV outcomes were, for the first time, assessed in post-menopausal diabetic women in primary prevention. The discovery of PCSK9 and the consequent approval of PCSK9 inhibitors has radically changed the management of patients at CV risk (31). In fact, PCSK9 has been recognized as a biomarker of CV risk in primary and secondary prevention by recent epidemiological studies (24, 27, 32), although its role has to be fully investigated in different cohorts of patients at CV risk, such as post-menopausal diabetic women.

In the present study, median serum PCSK9 levels were slightly higher than those observed in previous studies (24, 33) probably due to the characteristics of the enrolled patients. Indeed, several studies confirmed higher PCSK9 levels in patients with type 2 diabetes mellitus (T2DM) than the general population (34, 35) as well as in women than in men (11, 33). Moreover, higher baseline PCSK9 levels were observed in females with new diagnosis of T2DM than in those early affected by T2DM (36). Differences in PCSK9 concentrations were also detected between post-menopausal and pre-menopausal women (11, 37), as a consequence of the estrogen decrease related to menopause (8). Indeed, high levels of estrogens significantly reduce PCSK9 levels with the consequent increase of LDLRs in liver (38). In addition, the increased levels of PCSK9 may reflect the reduction of PCSK9 clearance mediated by LDLRs reduction consequent to the decreased estradiol levels (39) following menopause, as previously mentioned. The relationship between estradiol and PCSK9 is affected by transcriptional and post-transcriptional mechanisms through an estrogen receptor *α*-mediated pathway (40) as well as through the G-protein estrogen receptor activation (41) and LDLR mRNA expression (42).

In our study, women in late-onset menopause stage showed significantly reduced PCSK9 levels compared to women in normal-onset menopause. Moreover, late-onset menopause stage was inversely related to PCSK9 concentration. This data could be explained by the prolonged exposure to physiological estrogens that have protective and beneficial effects (11). No significant differences were observed between women in early- and normal-onset menopause stage and this was in contrast to a previous study showed that PCSK9 levels were not affected by estrogen replacement therapy in postmenopausal women (*p* = 0.6). This data support the hypothesis that menopause affects PCSK9 levels independently of estrogen therapy (39), PCSK9 clearance and LDLRs (43). Nonetheless, sex hormones might influence PCSK9 levels in post-menopausal women affected by diabetes and an increased prevalence of diabetes was observed in early-onset menopause patients compared to women in normal-onset menopause (44).

In contrast with different studies (24, 36), LDL-C values were inversely related to PCSK9 concentration in our cohort of patients. Although a correlation of PCSK9 plasma concentrations and LDL-C was previously described, LDL-C changes do not necessarily result in a respective modification of PCSK9 levels (39). The mutual correlation between PCSK9 and LDL-C is the result of a complex series of events that influence their plasmatic levels (45). In fact, when LDL-C is high, LDLR number would increase, also due to PCSK9 reduction, thus augmenting, plasma LDL-C clearance. On the contrary, when LDL-C levels are low, more PCSK9 would be active, thus stimulating liver LDLR degradation and limiting LDL-C clearance (46). In a previous study, no association was observed between PCSK9 and LDL-C in diabetic patients (47), but an effect of estradiol on the inverse correlation between LDL and PCSK9 cannot be excluded. Indeed, estradiol plasma levels may influence the correlation between PCSK9 and LDL in women (43); a negative correlation between estradiol and LDL-C adjusted for PCSK9 confirmed estradiol effects on LDL-C independently of PCSK9 (8). Indeed, the link between low PCSK9 levels or its inhibition and LDL-C levels with diabetes suggests a more complex interaction as Mendelian randomization analyses were almost concordant in showing an increased risk of new-onset diabetes in patients treated in the FOURIER and ODYSSEY trials (48). Therefore, our results confirm the possible correlation between PCSK9 and LDL-C levels also in a population of post-menopausal diabetic women.

The altered LDLR pathway clearance of plasma LDL-C may be one of the main causes of hypercholesterolemia that may also occur for mutations of LDLR and APO-B100; also, PCSK9 gain-of-function mutations may be associated with hypercholesterolemia whereas loss-of-function mutations are responsible for lowered plasma LDL-C levels, thus decreasing CV risk. In this study, we found that ApoB, which is the LDL protein component that binds LDLR, was an independent predictor of PCSK9 concentration, and as ApoB increases, an increase of PCSK9 may be detected in post-menopausal women with diabetes. Post-menopausal diabetic women show an increase of PWV values, in fact both menopause and T2DM were significantly associated with increased PWV (49). The relationship between CV risk factors and endpoints showed that PCSK9 levels influence PWV in post-menopausal diabetic women, as already observed in previous studies that demonstrated this correlation in different cohorts of patients (24, 26, 34). PCSK9 inhibitors effectiveness in reducing PWV confirmed the obtained data which also suggest the possible role of PCSK9 as a promising biomarker of CV risk and as a predictor of atherosclerosis independently of menopause (50) and lipid profile (51).

FPG is directly related to PWV as reported in other studies (52). Post-menopausal women with T2DM show increased PWV values, and both menopause status and T2DM are associated with PWV (53). This condition reflects the decreased arterial elasticity observed in diabetic patients because of oxidative stress, inflammation and advanced glycation end products that affect vessel wall (49). The passive stretching of collagen fibers not only may be responsible for PWV but also for PP changes and data obtained from other studies showed that SBP is positively associated with PWV and PP (34), as confirmed by our results; on the contrary, DBP is indirectly associated with PWV and PP.

Among the other parameters associated with CV risk, BMI was inversely related to PWV although the relation between BMI, obesity, and PWV is still controversial. In a study conducted on healthy subjects, BMI was negatively associated with PWV when adjusted for age, blood pressure, and additional CV risk factors. These data could suggest a possible benefit of obesity to arterial stiffness explained by the obesity paradox mechanism (54) and the smaller aortic diameter observed in lean individuals with the consequent increase of PWV values could further confirm this weak negative association (55). In addition, an inversely association was found between HDL and PWV values: the relationship between the lipid profile and arterial stiffness was evaluated by different studies although the results are discordant. High HDL values were related to increased arterial stiffness in post-menopausal women (56), but PWV was inversely related to LDL values in a population-based studies (57) as well as an independent, inverse relationship between HDL levels and PWV was observed in a cohort of healthy post-menopausal women (58).

## 5 Conclusion

In conclusion, our study demonstrated that PCSK9 levels increase in post-menopausal women and, specifically, the late onset stage menopause might influence both PCSK9 levels and consequently CV risk. The reported results further support the important correlation between PCSK9 and CV risk related to diabetes and menopause, thus highlighting its potential role as an additional biomarker of CV risk in post-menopausal diabetic women. Moreover, the obtained results might allow us to hypothesize that PCSK9 might be considered as a therapeutic target in a future clinical scenario. However additional validation studies are needed to validate our results.

## Data Availability

The raw data supporting the conclusions of this article will be made available by the authors, without undue reservation.
